# Improving the Efficiency and Quality of Sustainable Industrial CT by Optimizing Scanning Parameters

**DOI:** 10.3390/s25082440

**Published:** 2025-04-12

**Authors:** Íñigo Fonfría, Ibon Holgado, Naiara Ortega, Ainhoa Castrillo, Soraya Plaza

**Affiliations:** 1 Aeronautics Advanced Manufacturing Center, CFAA (UPV/EHU), Bizkaia Technology Park, Building 202, 48170 Zamudio, Spain; ibon.holgado@ehu.eus (I.H.); ainhoa.castrillo@ehu.eus (A.C.); 2 Department of Mechanical Engineering, Bilbao School of Engineering, UPV/EHU, Plaza Ingeniero Torres Quevedo 1, 48013 Bilbao, Spain; naiara.ortega@ehu.eus (N.O.); soraya.plaza@ehu.eus (S.P.)

**Keywords:** X-ray computed tomography, sustainability, energy consumption

## Abstract

Industrial Computed Tomography (CT) is a widely used Non-Destructive Testing (NDT) technique for evaluating internal and external geometries with high accuracy. However, its integration into industrial workflows is often hindered by long scan times and high energy consumption, raising sustainability concerns. This study introduces a novel approach to improving CT efficiency by integrating real-time energy consumption monitoring into the scanning process. A power measurement device was used to correlate scan parameters with energy usage and image quality, enabling a data-driven approach to parameter optimization. Results show that higher voltages improve image quality up to 32%, when evaluated using Contrast-to-Noise Ratio (CNR) amongst other image quality metrics, while reducing overall energy consumption by up to 61%. The results presented support the optimization of CT scan parameters by providing quantitative guidelines to balance efficiency, image quality, and sustainability. Additionally, deviations in dimensional measurements obtained through CT scans were compared against reference data from a Coordinate Measuring Machine (CMM), with differences up to ±45 μm. The findings contribute to enhancing CT performance while minimizing environmental impact.

## 1. Introduction

Computed Tomography (CT) is becoming a remarkable solution in the Non-Destructive testing (NDT) field, especially in the analysis of different materials and in the inspection of complex geometries [1]. Its ability to provide detailed information about both internal and external characteristics of a sample without direct contact makes it applicable in a wide variety of fields, including the aerospace, aeronautics, and automotive industries.

Although there have been various attempts to understand and measure individual sources of error in the CT process, no standardized method has been established to accurately evaluate the uncertainty of its dimensional measurements. Currently, only drafts, guidelines, descriptions, or definition documents can be found, with experimental methods being the most used approaches for estimating CT measurement uncertainties. Notably, the VDI/VDE 2630-2.1 guidelines [2] focus on applying the empirical substitution method to CT measurements, a method frequently implemented in the literature. The substitution method is performed by means of so-called reference artefacts, which adapts ISO 15530–3 for tactile Coordinate Measuring Machines (CMM) to CT [3]. Importantly, it does not necessitate an explicit evaluation of measurement uncertainty for each individual source of error. There is a scientific consensus that the use of artefacts represents a widely employed solution for verifying and calibrating CT measurement processes.

In parallel, the rise of additive manufacturing (AM) has introduced new challenges and opportunities in industrial CT. AM enables the fabrication of intricate geometries that are otherwise impossible to achieve using conventional manufacturing methods. One of the most critical applications of AM is in heat exchangers, where optimized designs improve thermal performance while reducing weight, a key factor in aerospace applications. Careri et al. pointed out that the fabrication of heat exchangers through additive manufacturing enhances their efficiency, as it enables the creation of complex internal geometries that optimize heat transfer [4]. Nevertheless, the internal complexity of the AM manufactured parts implies a challenge for quality inspection, since conventional metrological technologies are not enough to evaluate their dimensional accuracy and integrity because they cannot physically access those internal areas. In this context, CT remains the most suitable method for inspecting AM components due to its ability to capture internal details. Regarding the materials used in the additive manufacturing of heat exchangers, aluminum, stainless steel, and titanium emerge as the most commonly employed options. These materials are chosen for their great thermal and mechanical properties in demanding applications.

Beyond the need for accurate inspections, efficiency has become a crucial factor in CT applications. In today’s industrial landscape, the need to reduce scan time and energy consumption is imperative to align with sustainability goals. Shorter scan times enhance production and are supportive in reducing costs whilst also addressing sustainability by less energy use and low environmental effect. With this information, the challenge now lies in speeding up scan times without compromising data quality and, at the same time, avoiding significant increases in energy consumption.

Among these complexities, it cannot be overlooked that parameters are important regarding performance in CT. Parameters such as voltage, tube current, or exposure time have their influence on the scanning duration, the quality of the data obtained, and the energy expended. Understanding the interplay of these parameters is a crucial step towards optimizing the potential of CT for Non-Destructive Testing and for accurate dimensional measurements.

Various approaches have been developed regarding the CT scanning process optimization challenge. In this context, Reiter et al. [5] propose a simulation-based approach to optimize CT scanning parameters. The findings demonstrate how simulation may predict optimal parameters before actual scans, also reducing the need for physical tests, enhancing industrial CT’s sustainability. Nevertheless, simulating CT processes is a complex task, since they are influenced by a large number of factors making it difficult to achieve realistic data.

An alternative approach is presented by Bellens et al. [6], in their review on the application of machine learning techniques in industrial CT. The authors describe how Artificial Intelligence-based machine learning techniques may determine optimal acquisition parameters and scanning trajectories by learning from previous successful scans. Data-driven approaches enable the analysis of complex relationships between material properties, geometrical features, and scanning parameters. Conventional methods might overlook these interactions. This approach enhances efficiency by eliminating the need for trial-and-error testing.

However, in spite of the advances in parameter optimization, the literature shows a gap regarding the actual measurement and analysis of CT energy consumption. Addressing this gap is essential as energy efficiency has become a crucial aspect in industrial environments. For instance, although not explicitly demonstrated in industrial CT applications, recent research by Fenerich et al. [7] provides an updated review of energy efficiency in industrial settings, highlighting strategies used to reduce energy consumption while keeping quality up.

The primary objective of this study was to evaluate the impact of CT parameters such as voltage, step size, and the number of radiographies per step (RPS) on scan time, data quality, and energy efficiency, using real energy consumption data. This work focuses specifically on enhancing performance in NDT inspection by obtaining high-quality measurements without excessively increasing energy consumption, while quantifying the relationship between these factors. By systematically analyzing these parameters, this study aims to identify those with the greatest influence and optimize their configuration for improved overall performance.

Additionally, this research emphasizes energy efficiency through detailed consumption measurements for CT procedures, making it particularly relevant in understanding the environmental impact of CT inspections. This approach aligns with the United Nations Sustainable Development Goals, which include ensuring sustainable consumption and production patterns [8].

To sum up, the goal is to facilitate informed decision-making in the setup of CT parameters, advancing the field of Non-Destructive Testing and dimensional measurement in terms of both accuracy and sustainability.

## 2. Materials and Methods

### 2.1. Set-Up

Specimen preparation and measurement equipment set up are key points in ensuring high-quality tomographic data. This section outlines the details pertaining to the specimen under investigation and the instrumentation employed in this study. 

The quality of the tomographic data is dependent on how effectively the specimen is prepared and how correctly the measuring equipment is set up. This section covers the specifics related to the specimen in question and the equipment used in this research.

#### 2.1.1. Specimen

**Figure 1:**
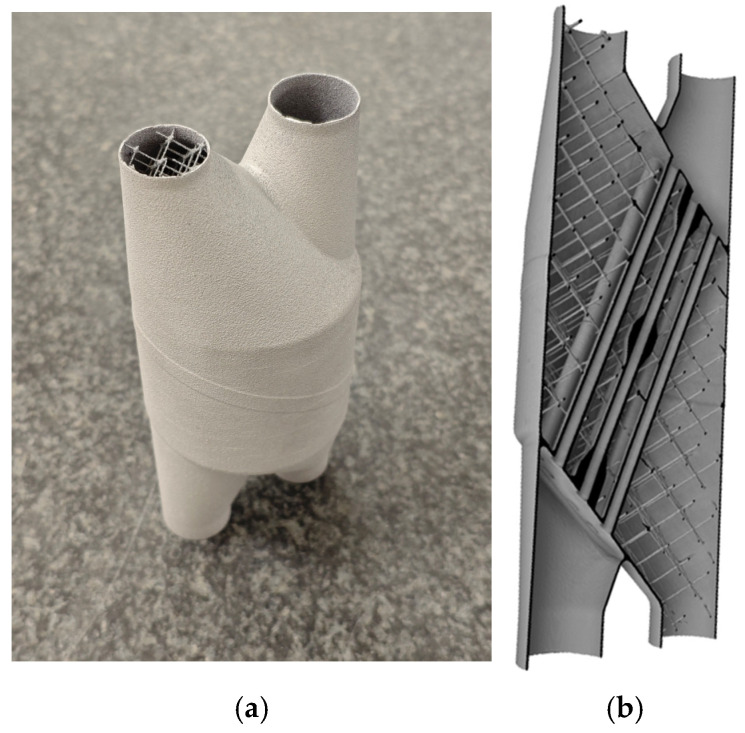
Heat exchanger specimen. (**a**) Physical part used for testing and (**b**) cross-sectional view of the CAD model revealing internal geometry.

This section provides details concerning the specimen being analyzed as well as the actions undertaken to conduct this study. The object under inspection is a heat exchanger fabricated by AM characterized by an intricate geometry, as illustrated in Figure 1.

AM offers a wide variety of techniques for producing metal components, including Binder Jetting (BJT), Directed Energy Deposition (DED), and Material Jetting [4]. However, the method used in this study, and the most widely adopted in industry, was Laser Powder Bed Fusion (PBF-LB), due to its ability to produce high-integrity parts with excellent mechanical properties.

The specimen used in this study was selected to represent a class of industrial components where both geometric complexity and material properties played a critical role in performance and manufacturability.

The heat exchanger design was inspired by shell-and-tube configurations, where one fluid flows through a network of internal tubes while another passes through the surrounding shell. The shell incorporates a lattice structure that enhances heat transfer efficiency by promoting turbulent flow. Given its complex internal geometry, CT is the only viable method for conducting accurate dimensional inspections of such components, reinforcing the relevance of using this specimen in this study.

Heat exchangers are widely used in industries such as aeronautics, thermal systems, and Heating, Ventilation, and Air Conditioning due to their efficiency in transferring heat between fluids while maintaining separation. In many applications, the working fluids can be chemically aggressive, requiring materials with high corrosion resistance to ensure durability and operational reliability. For this reason, S31673 stainless steel was chosen for manufacturing the specimen. This material is preferred in heat exchanger construction because it resists chloride-induced corrosion and oxidation, making it suitable for environments such as seawater cooling systems, chemical processing, and food production. Its mechanical properties and resistance to harsh conditions extend the lifespan of these components while reducing maintenance costs.

Additionally, S31673 stainless steel was selected over alternatives like aluminum because its higher density is expected to result in greater energy consumption during CT scanning. This characteristic made it a more suitable candidate for evaluating optimization strategies aimed at reducing energy use while maintaining scan quality.

Regarding the relevant dimensions under study, the heat exchanger featured thirteen parallel central fluid channels with a nominal internal diameter of 3 mm. Apart from that, the dimensions of the bounding box enclosing the sample were an external diameter of 60 mm and a height of 150 mm. These dimensions ensure that the entire component can be scanned in a single scan, considering the size constraints of the detector of the equipment.

**Figure 2:**
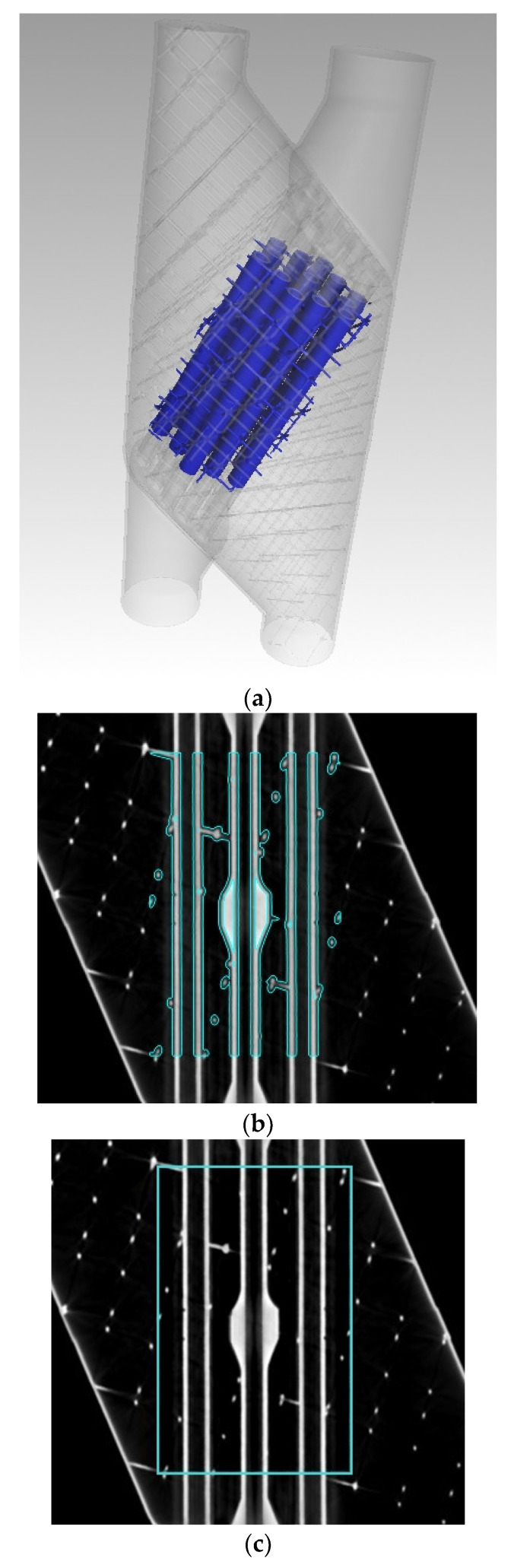
Regions of Interest for the analysis of the heat exchanger. (**a**) Complete central zone including the thirteen fluid channels (without transparency highlighted in blue); (**b**) whole central region encompassing the thirteen fluid channels plus the air among them (ROI 1); and (**c**) 1.6 mm thick region following the surface boundaries between material and void (ROI 2).

Subsequent analyses were conducted on specific cylindrical Regions of Interest (ROI) of the specimen. The chosen regions enclose the central section of the heat exchanger, as shown in Figure 1 encapsulating the thirteen central fluid channels in a 23 mm diameter and 50 mm height cylinder. One region, defined as “ROI 1”, encompassed the entire central zone of the specimen, including all thirteen fluid channels plus the air among them, while the other, “ROI 2”, focused solely on areas delimited by the surface of the piece. This may be seen in Figure 2. Specifically, it consists of a 1.6 mm thick region that involves the area where the specimen’s surface was defined. This region covers the entire delineated surface of the tubes, indicating the boundary between material and void. A 1.6 mm thickness was chosen as Du Plessis et al. [9] proposed to use a 5-voxel span from each side of the surface to carry out the relevant analyses.

#### 2.1.2. Measuring Equipment

The CT inspections featured in this study were conducted at the Aeronautical Advanced Manufacturing Centre (CFAA) affiliated with UPV/EHU, utilizing the digital radiography system X-CUBE compact 225 (General Electric, Boston, MA, USA). Once the tests were completed, the post-processing was carried out using the VGStudio MAX 2023.3 software (Volume Graphics GmbH, Heidelberg, Germany).

**Figure 3:**
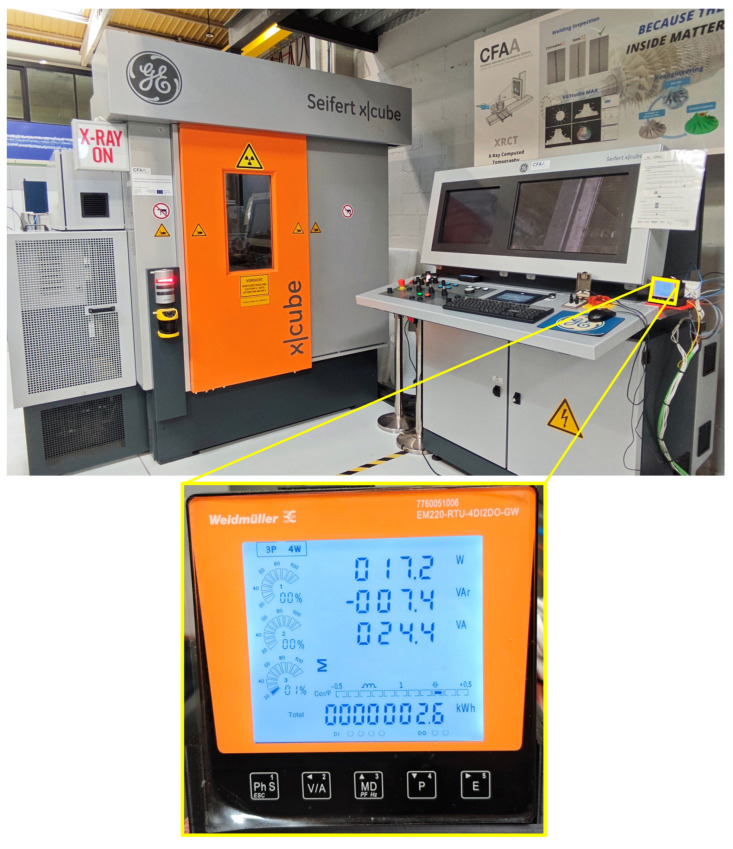
Power measurement device (Weidmüller EM220-RTU-4DI2DO-GW) integrated in the CT equipment.

Energy consumption data acquisition was carried out using a power measurement device, namely the EM220-RTU-4DI2DO-GW (Weidmüller Holding AG & Co. KG, Detmold, Germany) [10]. Figure 3 shows the integration of both systems configured for simultaneous CT inspection and power consumption data acquisition.

This device is a three-phase power meter that is integrated into the system by a three-phase AC supply. It is a multipurpose device that is able to measure and monitor several electrical parameters, such as active, reactive, and apparent power, power factor, and frequency besides the more usual voltage and current. It also features an IP51 rating, providing protection against dust and contact, guaranteeing a proper functioning within the working environment.

Regarding its technical specifications, it includes a voltage measurement range of 75–270 V AC and 100–380 V DC, frequency selection of 50/60 Hz, and active power measurement accuracy of 0.5% (i.e., Class 0.5S as certified by IEC 62053-22). The instrument also has the capacity to obtain real-time data after every one second of updates, making sure that there is uninterrupted monitoring.

This device was used because of its ability to measure the active unitary power consumption of the equipment while scanning, making it possible to obtain the total energy consumed during each scan based on these measurements.

### 2.2. Methodology

**Figure 4:**
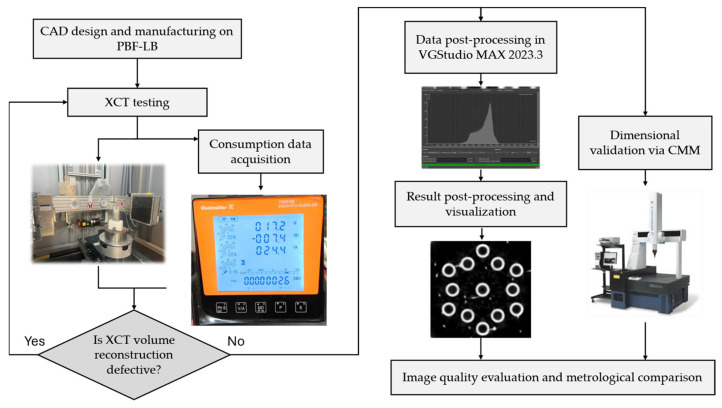
Schematic representation of the research methodology.

Here, the procedure followed during the tests, the parameters used for each of them, and the results obtained after post-processing are outlined. The way the procedure was conducted is shown in Figure 4.

#### 2.2.1. Procedure

The procedure described in this work follows a series of straightforward and sequential steps to achieve the desired outcomes. Firstly, in the initial phase, the set-up of the specimen inside the working area of the CT system took place. The positioning of the specimen was carefully carried out ensuring it can be scanned entirely in one single operation, which was essential to optimize data acquisition. The magnification level was selected so a voxel size of 0.155 mm^3^ was achieved. The voxel size resolution determines the smallest distinguishable feature in the reconstructed volume. A smaller voxel size improves spatial resolution, enhancing the ability to detect fine geometric details. A voxel size or resolution of 0.155 mm^3^ means that each side of the cubic elements forming the reconstructed volume of the specimen measures 0.155 mm. Simultaneously, the scanning parameters were established, and the power meter was set up to monitor and record energy usage data.

Real-time energy measurement plays a crucial role in continuously tracking power consumption during the CT scanning process. This capability is essential not only for optimizing machine performance but also considering new EU regulations requiring nearly all products to feature a Digital Product Passport (DPP). The DPP, part of the Eco-design for Sustainable Products Regulation, aims to enhance transparency throughout product value chains, covering aspects such as product origin, materials, environmental impact, and disposal recommendations [11]. In this context, real-time machine data extraction becomes a valuable tool for correlating scanning parameters with energy consumption, allowing for the dynamic optimization of the process. This capability provides immediate feedback on machine performance, enabling prompt decision-making, early anomaly detection, and continuous process improvement.

**Figure 5:**
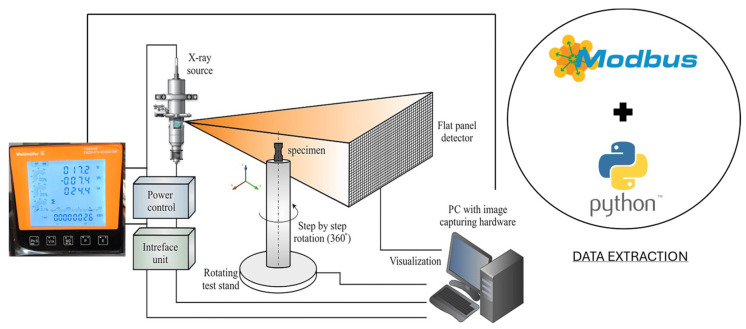
System architecture of the CT equipment with automated data extraction capabilities via Modbus protocol and Python scripting.

The energy usage data the device collects was retrieved using a Python script (version 3.11.9). Along with the script, Modbus Transmission Control Protocol (TCP) was utilized to establish communication with the power sensor. Modbus TCP protocol is commonly used in industrial automation because it reliably enables data exchanging between devices. This protocol operates on a client-server architecture where the client (in this case, the Python script) sends requests to the server (the power sensor) to read or write data registers, allowing for efficient transmission of measurement values across ethernet networks without significant overhead, making it ideal for monitoring applications where consistent data acquisition is critical. In this case, a command-line tool named Modpoll was employed, which is capable of sending queries to the sensor over a local network. These queries were sent to request specific registers from the device, corresponding to specific parameters such as active power, voltage, or current, among others. Figure 5 illustrates the schematic overview of the integrated system for CT acquisition and power consumption data measurement.

**Figure 6:**
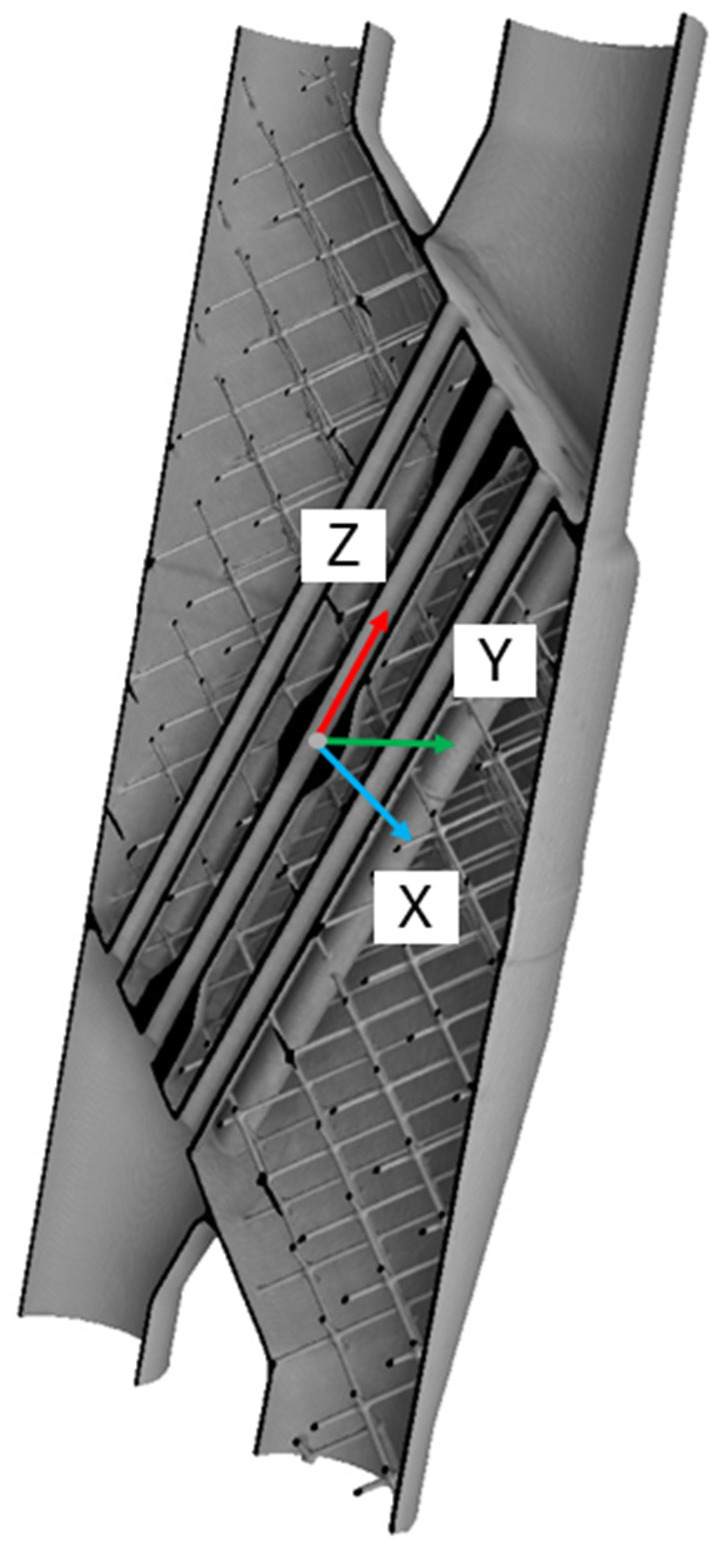
Alignment of the specimen, showing its reference system and internal structure.

When the data acquisition system was set up, the sample was sequentially scanned in the CT equipment, varying scanning parameters in a controlled way. Then, the volume of the specimen was reconstructed thanks to the Feldkamp–Davis–Kress mathematical reconstruction algorithm, preparing the sample to be analyzed with the VGStudio MAX 2023.3 software. The post-processing stage started by creating a surface representation of the specimen using an advanced local adaptive algorithm throughout the volume. Then, once the surface that sets the threshold between what is object and what is background was defined, an alignment of the volume was performed. The alignment involved setting the *Z*-axis along the central internal cylinder’s axis and then defining the *X*-axis as a projection of the line connecting the axes of the outer cylinders. The coordinate system may be seen in Figure 6. Following the same process along subsequent tests made subsequent analyses consistent; by making spatial references consistent and minimizing misalignment problems, spatial references were standardized across all samples, enabling direct comparisons and minimizing variability caused by misalignment.

Lastly, a grayscale analysis was conducted on the ROIs outlined in Section 2.1.1, with the purpose of extracting various noise and contrast metrics. The metrics used to evaluate image quality are shown below. These include the Contrast-to-Noise Ratio (CNR), Signal-to-Noise Ratio (SNR), and the quality metric (Q), which were selected based on their extensive use and standardization in CT imaging quality assessment [9]. These metrics provide a robust evaluation framework, covering different aspects of image quality such as basic signal fidelity (SNR), contrast enhancement (CNR), and statistical properties of the reconstructed volume (Q metric) [12]:

1. Contrast-to-Noise Ratio (CNR):

The Contrast-to-Noise Ratio (CNR) is one of the most important metrics for characterizing the degree of visibility of the depicted object against its background in the image. This metric, as defined by ISO 15708-2 [13], offers valuable information about image quality, specifically regarding how distinctly the contrast between different regions of interest can be distinguished [14]. Baraka et al. [12] emphasizes that CNR is essential in dimensional metrology, because it indicates that low values affect measurement uncertainty by reducing distinguishability between features.

The CNR equation is shown next:

(1) $$CNR=\frac{g_{object}-g_{background}}{\sigma_{background}}$$

This formula is applied to ROI 1, and $g_{object}$ represents the mean gray value of the region of interest corresponding to the material, while $g_{background}$ is the mean gray value of the background region (air). These gray values correspond to intensity levels in the histogram derived from the $2^{14}$-bit depth of the reconstructed image. The parameter $\sigma_{background}$ denotes the standard deviation of the gray values in the background region, serving as a measure of noise in the image.

2. Signal-to-Noise Ratio (SNR):

The Signal-to-Noise Ratio (SNR) is a basic measure that is expressed as the proportion of the signal contained in the image of the subject to the noise present in the image of the subject itself [14]. Baraka et al. [12] explains that SNR is a key metric when assessing the quality of 2D images extracted from the reconstructed volume under study. Industries benefit significantly from a high SNR as it enhances the fidelity of imaging results, contributing to more accurate analyses and decision-making processes. The formula defining this metric is shown next:

(2) $$SNR=\frac{g_{object}}{\sigma_{object}}$$

This metric also applies to ROI 1, and, in this case, $g_{object}$ also represents the mean gray value of the material region in the histogram. $\sigma_{object}$ is the standard deviation of these gray values again, indicating the noise level in the material itself.

3. Q metric:

The Q metric introduces a different approach to image quality assessment by utilizing the peak values of the grey value histogram. This metric, derived from scans and referenced in [15], measures the degree of grey value separation between different materials in the histogram, with a focus on background and part distinctions. The Q metric equation is:

(3) $$Q=\frac{\left|{µ}_{object}-{µ}_{background}\right|}{\sqrt{{\sigma_{background}}^{2}+{\sigma_{object}}^{2}}}$$

Here, ${µ}_{object}$ represents the mean value of the gray peaks in the material region of the histogram, while ${µ}_{background}$ corresponds to the mean value of the gray peaks in the background. The terms $\sigma_{background}$ and $\sigma_{object}$ are the standard deviations of the gray values in the material and background regions. To better understand this section, the method followed is the one Du Plessis et al. [9] outlines in his work, where a Region of Interest like ROI 2 is used selecting 5 voxels at each side of the surface where the grey value analysis is applied.

Finally, the data that were extracted from the tests underwent the last stage of processing in Python, resulting in the obtention of conclusions that contribute to the existing body of knowledge on the subject matter under investigation.

Additionally, with the intention of providing a quantitative validation of the CT measurements and their dependence on scanning parameters, a complementary analysis using a CMM was performed, Mitutoyo Crysta Apex S 9106 [16] being the equipment used for this task. This kind of equipment is widely used in the metrological field as it thoroughly follows international standards such as the ISO 15530 series [3], which provides methodologies to assign uncertainty to measurements. In contrast to CT, because of its adherence to these standards, direct uncertainty assignment is allowed with CMM technology. Thus, the methodology for the validation of CT measurements using CMM as a reference is widely used in the technical literature. For example, as Villarraga-Gómez [17] did, comparing internal features’ measurements.

**Figure 7:**
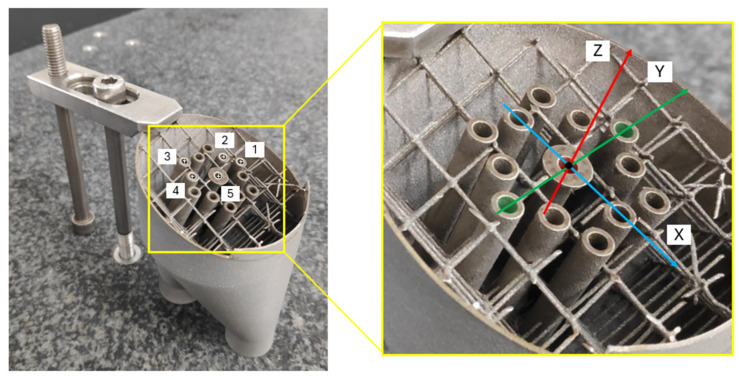
Workpiece setup on the CMM, showing the defined coordinate system and the measured holes.

Sample preparation entailed the use of wire Electrical Discharge Machining (EDM) to cut out a core section of the heat exchanger, orthogonal to its internal cylinder, while minimizing any mechanical distortion that may interfere with the measurements. The specimen had a reference mark that specifies the location of the central strip, which was useful to maintain comparability between the two techniques. Several dimensional and geometrical measurements were made at locations within this central region, specifically on the inner diameters of the internal cylinders, shown in Figure 7.

This way, it became possible to relate values from standard imaging quality parameters, such as CNR, SNR, and Q metric, with actual measurement accuracy, providing practical information on the CT equipment’s performance under varying scanning conditions.

The procedure of correlation between CT measurements and CMM measurements began with the creation of a comparable method to be carried out with both techniques. The method included a part alignment process capable of being executed equally by both techniques, and a measuring process of the same regions of the sample.

The tools used during the whole process in the CMM were the Renishaw SP25M scanning probe system [18] combined with the Renishaw A-5003-0577 touching probe [19]. Noteworthy is that the SP25M system implied using probe angle adjustments in 7.5-degree increments, as it does not allow to freely choose an exact orientation. The A-5003-0577 touching probe consists of a 0.7 mm ruby sphere tip mounted on a 20 mm WC stylus.

Five specific holes were measured, shown in Figure 7, and a measurement procedure was created focused on measuring 3 fundamental characteristics of the holes: the diameter, the roundness deviation, and the relative distances between holes’ centers. Each hole was measured by probing 12 equally distributed points inside the hole’s internal surface, forming a circumference, and every measurement was repeated 8 times for each hole. The measurements were taken at a −2 mm nominal depth from the origin of the coordinate system, due to the features of the piece and the measuring system.

Once the alignment process and the measuring process are completed in the CMM, the same procedures were replicated in VGStudio MAX to the CT scans, with the aim of enabling result comparison between both technologies.

First, the exact same alignment strategy was followed in CT in order to get the exact same reference system as in Figure 6, using the internal surfaces of the same central and lateral tubes to generate the cylinders that were used as datum features. The point-fitting method used to generate the cylinders was the maximum inscribed method for inner circles, the same way as it was performed in the CMM.

Using this auxiliary plane enabled finding the distance between the origin of the coordinate system and the support surface in the CMM. Consequently, that distance could be applied to the CT scans, this way, defining the exact same coordinate system in both technologies. Later, the same circle measurements as in CMM were performed. Hole diameters and roundness deviations were calculated.

#### 2.2.2. Design of Experiments

**Figure 8:**
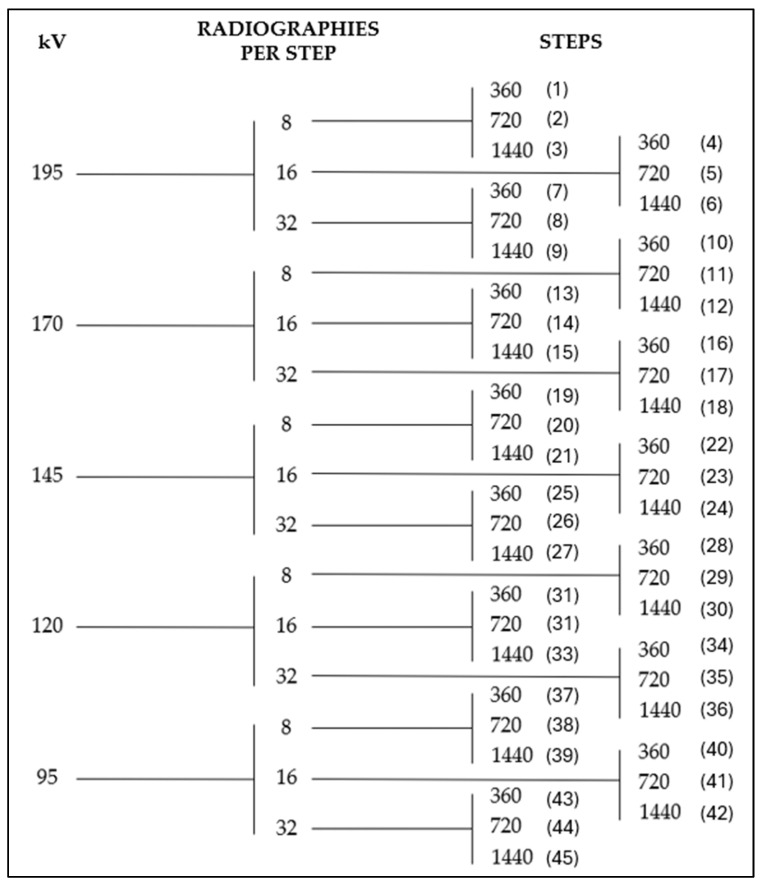
Parameter combination tree diagram.

A total of 45 tests were carried out combining the following parameters (Figure 8): voltage, RPS and steps. The scanning parameters were selected based on their relevance to image quality and scanning efficiency, as reported in key studies on industrial CT for metrological applications [20]. Voltage was chosen because it directly determines the energy of the X-ray photons, which affects their penetration capability. In turn, the step and RPS were selected due to their influence on the amount of data collected for image reconstruction, and therefore on the quality and duration of the scan. These parameters are particularly relevant when optimizing scanning strategies in terms of both quality and energy consumption [21].

It should be noted that other parameters, such as tube current or exposure time, also influence the process, but their adjustment is intrinsically linked to the voltage. For each CT scan, a specific voltage and a fixed value of tube current was chosen to make sure that the X-rays can penetrate the given specimen, and according to the manufacturer’s recommendations. The exposure time has been modulated so the grey colors per channel were established to a value ranging from 11,700 to 11,800 out of 16,384, which was the full amount of different grey colors of a 14-bit depth. Additionally, grayscale values were set within this range, representing about 70% of total depth. This setting allowed for post-processing with the widest possible grayscale range. It also prevented potential detector damage that could result from excessive X-ray energy.

In this work, specific voltage values were used for the tomography sessions, these being 95 kV, 120 kV, 145 kV, 170 kV, and 195 kV. These values stemmed from prior experiences conducted on similar S31673 stainless steel components to the specimen under study [22]. The voltage values were selected based on the upper limit of the CT equipment (195 kV), with the lower limit being the minimum value that guarantees proper X-ray penetration. The corresponding values of tube current and exposure time to meet the manufacturer’s recommendations for optimal scanning were: 2.6 mA in the case of the current and, for the exposure time, 33 ms in 195 kV scans, 45 ms in the case of 170 KV scans, 63 ms in the case of 145 kV scans, 107 ms in the case of 120 kV scans and 245 ms in 95 kV scans.

On the other hand, the steps were set to three different values: 360, 720, and 1440. The RPS directly determines the volume of data available for reconstruction. A higher RPS increases the amount of projection data acquired, which can improve image quality and the fidelity of the reconstructed model. In this study, three values, 8, 16, and 32, were selected to systematically evaluate this effect. The selection of both step values and RPS was first constrained by the lower operational limits of the CT system. To define the upper boundary, a maximum scanning duration of 3.5 h was established, as this was considered a reasonable limit for components of this size in industrial settings. Longer scan times were deemed impractical and incompatible with production-oriented CT workflows. This approach is also supported by the findings of Villarraga-Gómez et al. [21], who demonstrated that increasing the number of projections beyond a certain point yields negligible improvements in dimensional accuracy.

These parameter combinations resulted in 45 different scans, as shown in Figure 8. The figure presents all parameter combinations in a tree diagram format, with the test ID corresponding to each specific configuration displayed in parentheses at each terminal branch.

Repetitions of each scan ID were not performed due to resource constraints. However, to assess measurement stability, repeated scans were conducted for ID 5 configuration using the same CT equipment. From these repeated measurements, image quality parameters were calculated, yielding standard deviations of 0.001 for CNR, 0.027 for SNR, and 0.003 for Q metric. These minimal variations demonstrate the high repeatability of the measurement process, supporting the reliability of the results presented in this study and justifying the decision not to perform repetitions for all scan configurations.

## 3. Results

Within this section, the results of the experiments are examined, with a focus on establishing connections between image quality enhancement and the corresponding increase in energy consumption caused by the modification of input parameters. As it is previously said, the major concern was to establish a proper balance between energy consumption and image quality to increase efficiency without compromising environmental sustainability objectives.

Table 1 shows the mean values and standard deviations (Std. Dev.) for each output parameter, calculated based on voltage variations. The four output parameters mentioned above are presented to evaluate both quality and power (P) consumption across the test configurations. For detailed visualization, several individual scan results are portrayed in subsequent figures where specific examples are graphically represented.

**Figure 9:**
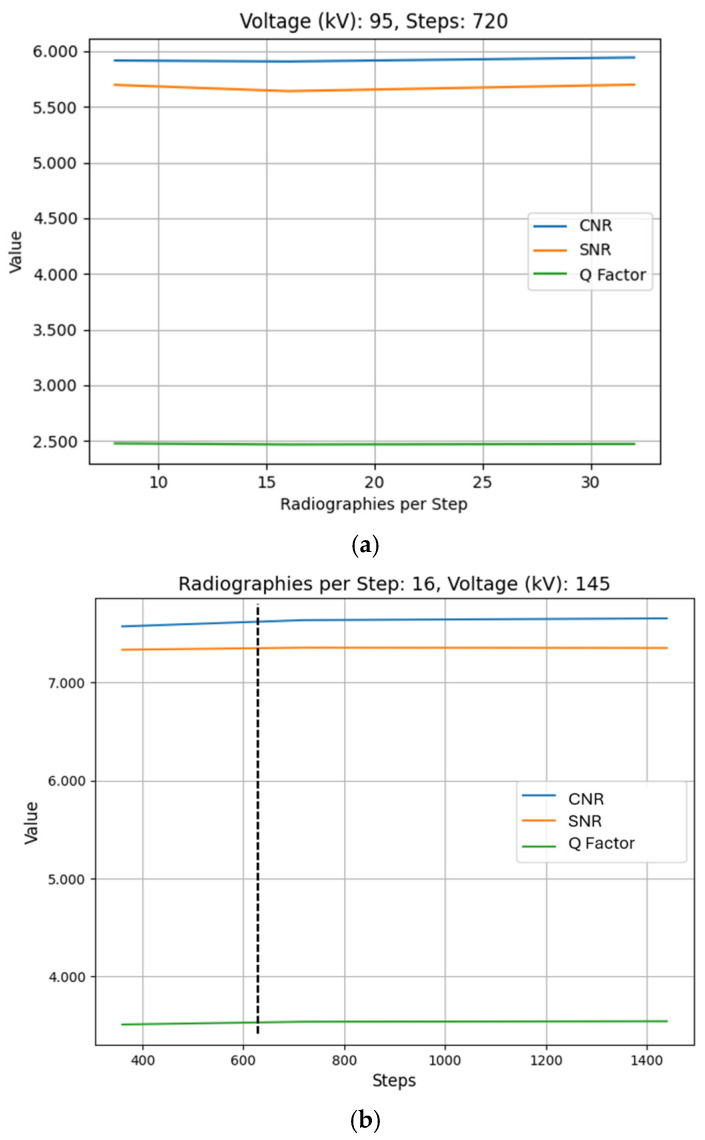
Comparison of quality metrics variations as a function of (**a**) RPS (Test IDs 38, 41, and 44) and (**b**) number of steps (Test IDs 22, 23, and 24).

After conducting the experiments and analyzing the obtained results, although some variation is observed across different scan steps and RPS values, the changes in image quality metrics (CNR, SNR, Q) remain below 2% of their corresponding values. As illustrated in Figure 9, varying these parameters shows that the quality parameters remain virtually constant across different combinations. The graphs for quality metrics against every parameter combination were generated. And, after observing the similarities, the graphs corresponding to test IDs 38, 41, and 44 in the first place, and 22, 23, and 24 in the second place are shown:

Each number of steps generates a displacement of the outer part of the specimen depending on the rotated angle and the dimensions of the specimen. A dotted line has been added with the purpose of indicating the value of steps needed for the specimen to fulfill a displacement equal to the resolution of the scans, which is a voxel size of 0.155 mm^3^. The number of steps needed to achieve that displacement is, specifically, 612.76 steps.

**Figure 10:**
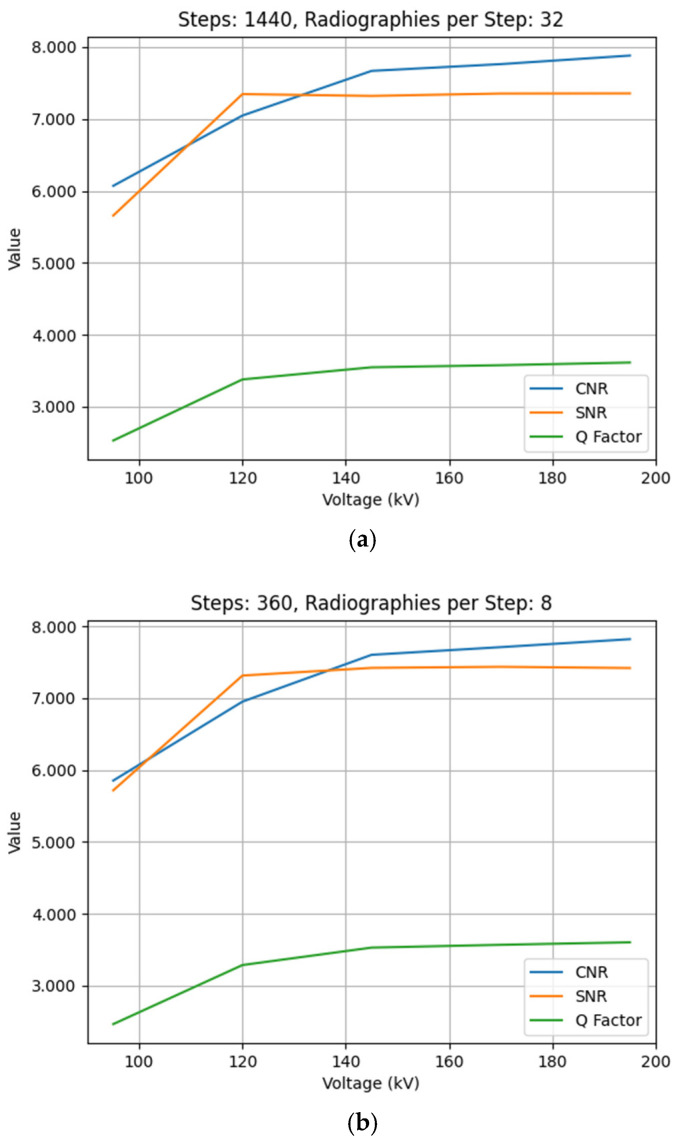
Comparison of quality metrics variations as a function of voltage (kV) for two configurations: (**a**) 1440 steps and 32 RPS (test IDs 9, 18, 27, 36, and 45); (**b**) 360 steps and 8 RPS (test IDs 1, 10, 19, 28, and 37).

Moreover, the impact of voltage variation on image quality was assessed and deeper understanding was gained. The results indicate a uniform improvement across all quality metrics in relation to the increase in the voltage values. As demonstrated by the graphs shown in Figure 10, where, again, after observing the similarities between the obtained graphs, the graphs corresponding to test IDs 9, 18, 27, 36, and 45 in the first place, and 1, 10, 19, 28, and 37 in the second place are shown in order to demonstrate the pattern. 

One notable observation, in addition to the consistent quality enhancement across all parameters as voltage increases, is that not all parameters follow the same pattern. The CNR parameter exhibits the greatest sensitivity and variability, as it demonstrates the largest value increase. Moreover, it is evident that CNR experiences more substantial increments in the lower voltage transitions (95 to 120 and 120 to 145 kV). For the higher voltage increments (145 to 170 and 170 to 195 kV), the CNR increase is more modest. In contrast, the Q metric exhibits the least sensitivity to voltage variations, following a similar pattern. In the case of SNR, a substantial increase is observed in the transition from 95 to 120 kV; however, the increase gets milder for subsequent voltage increments.

**Figure 11:**
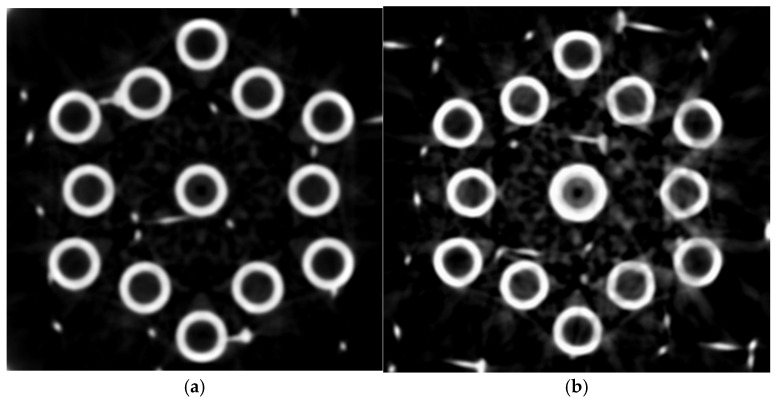
Visual comparison of the reconstruction obtained from VGStudio MAX 2023 as a function of voltage (kV): (**a**) scan at 195 kV; (**b**) scan at 95 kV.

A qualitative comparison between two different voltage level XCT images is shown in Figure 11, where the improvement in image quality can be attributed to the greater X-ray penetration capacity of higher voltage configurations.

After analyzing the influence of different parameters on image quality, a detailed study of the relationship between CNR and voltage was conducted, as voltage demonstrated to be the most influential parameter on quality metrics. The following graph presents the average CNR values obtained for each voltage level. It also shows a trendline adjustment of the curve to an exponential curve fit, due to the previously seen trend of the CNR values reaching a plateau meaning that X-ray penetrability is maxed out.

**Figure 12:**
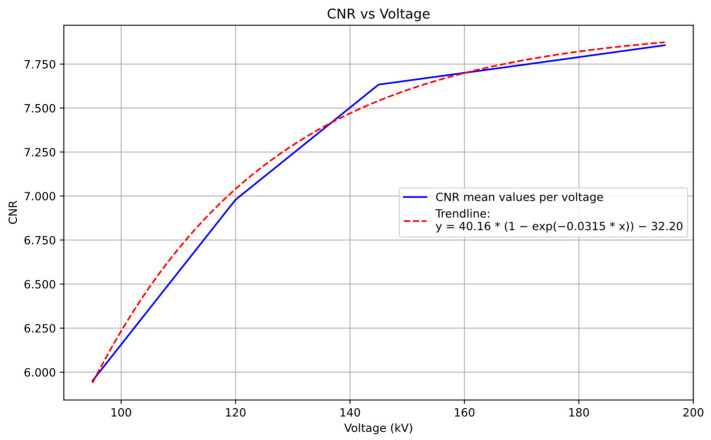
Average CNR values for used voltage (kV) values with an exponential fit trendline.

The graph in Figure 12 depicts the evolution of CNR as a function of the applied voltage, where each data point represents the average CNR value for a given voltage level. CNR rises with voltage, but the rate of increase diminishes at higher voltages. CNR exhibits substantial increments of 17.273% and 9.379% for the voltage transitions from 95 kV to 120 kV and 120 kV to 145 kV, respectively. However, for the higher voltage increments from 145 kV to 170 kV and 170 kV to 195 kV, the CNR improvements are relatively modest, at 1.457% and 1.452%, respectively.

Further analysis was conducted on the relationship between CT image quality and energy consumption. A new graph is shown below illustrating the connection between applied voltage, working power, and overall energy consumption. As may be seen, time is the main influencing parameter of total energy consumption, with all voltage slopes being remarkably similar. However, subtle increases in slopes are noticed as voltage progressively increases, indicating a slight rise in the equipment’s unit working power. Anyways, these changes do not represent a substantial influence on overall energy consumption.

**Figure 13:**
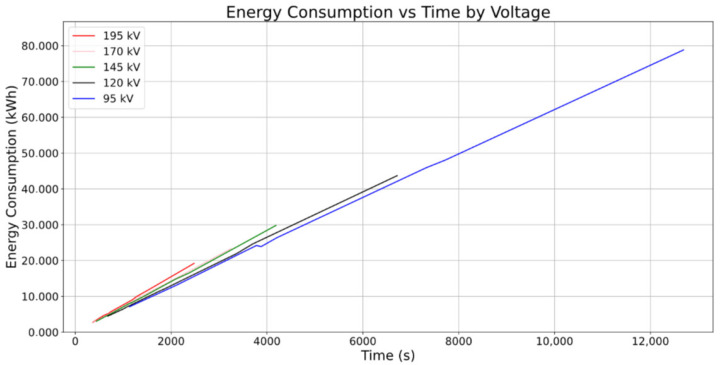
2D representation of the interactions between the following results on a voltage (kV) basis: overall energy consumption (kWh), and scan time (s).

The graph in Figure 13 depicts the connection between applied voltage, working power, and overall energy consumption. Notably, although increasing the input voltage raises the unit power consumption (as evidenced by the steeper ascending directions of the data points for each voltage in the Time vs. Energy Consumption plane), it also shortens the exposure time needed considerably. This phenomenon is evident in the graph, where at higher voltages, although unit consumption is greater, the overall energy consumption is much lower.

**Figure 14:**
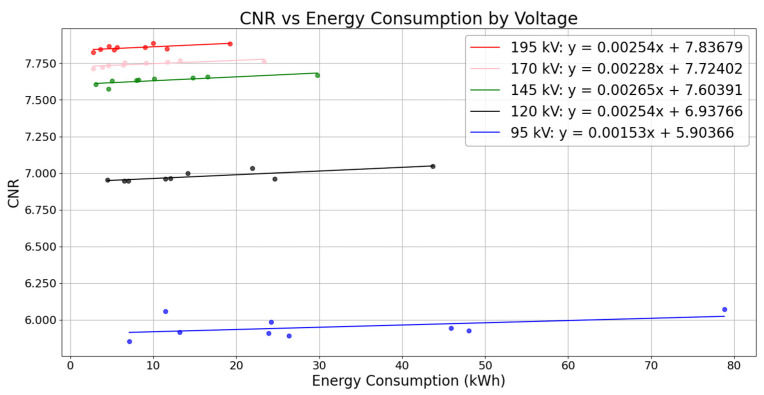
Comparison of CNR variations as a function of overall energy consumption (kWh) for each voltage (kV).

Going on with the results analysis, Figure 14 presents the relationship between CNR and energy consumption for each voltage value. The data show that, although higher energy consumption yields an improvement in CNR for every voltage value, that increment is very slight. The graph displays distinct curves for each voltage value, with multiple data points representing different exposure time configurations.

In the other hand, regarding the measurements made on the sample both on CMM and CT, the results obtained are shown in the following tables. The CMM measurement results are displayed first, in Table 2, showing the mean values and standard deviations of diameter (D) and roundness deviation (Rd. Dev.) for each hole, calculated from 8 measurement repetitions.

Next, the results obtained from the CT measurements are displayed, mainly showing the diameter and roundness deviation of each hole, in Table 3 and Table 4 respectively. The CT tests used for this task were the ones with the following IDs: 5, 14, 23, 32 and 41. These specific tests were chosen because voltage was identified as the most influential parameter, so varying the voltage while keeping the other parameters constant was preferred. All circles were obtained using the Gauss (least squares) method.

Before continuing, it is important to note that it was not possible to obtain the values from CT test 41 because, due to the lack of penetration capability of the X-rays at 95 kV, VGStudio MAX surface definition tool was not able to detect any surfaces in the inside part of the central hole. Therefore, making it impossible to align the part following the same method as in the CMM.

Derived from the data presented in the tables, several comparative analyses were carried out between the data extracted from the CMM and the data extracted from the CT scans. The objective of these analyses was to focus on the degree of agreement between the two approaches, focusing on the discrepancies or similarities of the extracted values.

**Figure 15:**
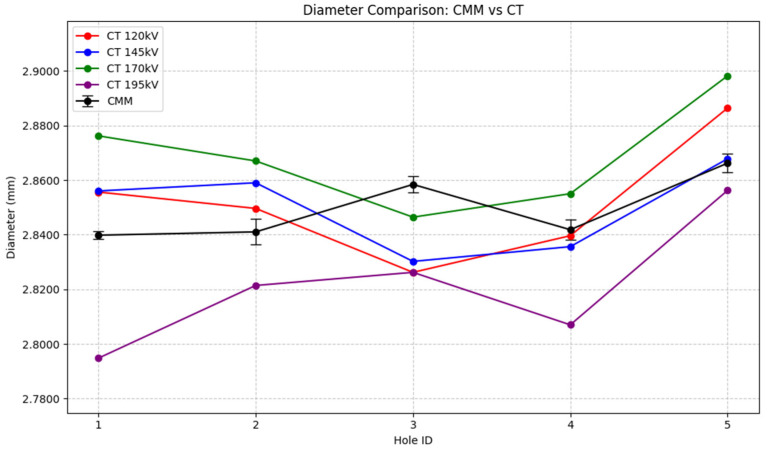
Comparison of the diameters (mm) obtained from the CMM vs the CT scans.

Firstly, the comparison between the hole diameters was conducted. The comparison is displayed in the following graph:

The diametral measurements performed on the 5 holes in both techniques are illustrated in Figure 15. At first glance, a nuanced pattern may be seen. Notably, the values obtained from the 120, 145, and 170 kV CT scans show an almost identical trend amongst them. Indeed, the mentioned trend also aligns closely with the CMM measurements, with the exception of hole 3. However, the 195 kV CT scan shows a different behavior. It matches the trend of the CMM measurements more closely, with the exception of hole 1.

**Figure 16:**
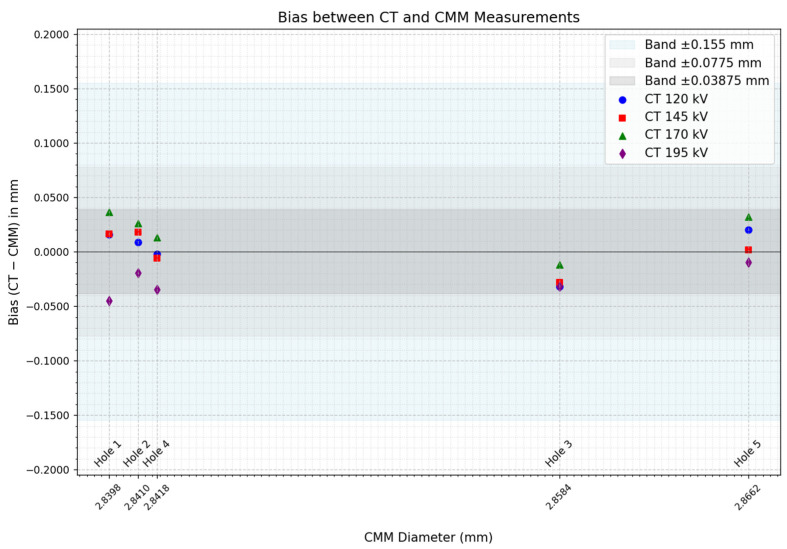
Absolute bias between the CT scans and the CMM diameter values (mm), compared to voxel size percentages (100%, 50%, and 25%).

In order to provide more information about the magnitude of the systematic differences between the two measurement techniques, the absolute bias between CMM and CT measurements was calculated. The results are shown in Figure 16:

After calculating the bias between the measurements of both techniques, it could be observed that a similar trend to the one in Figure 15 appeared. In addition, calculating the bias also made it possible to quantify the magnitude of the measurement discrepancies between techniques. The maximum error detected is approximately 45 μm, which is notably 4 times smaller than the 0.155 mm^3^ voxel size of the tomographic scans. This indicates that, if discrepancies remain below voxel size, CT scanning lacks sensitivity to reliably detect subtle geometric variations like the ones in the examined features. Furthermore, the graph also includes reference bands corresponding to the voxel size, 50% of the voxel size, and 25% of the voxel size. Notably, all values fall within the voxel size limit but even within 50% of the voxel size, further reinforcing the resolution limitations of the technique.

**Figure 17:**
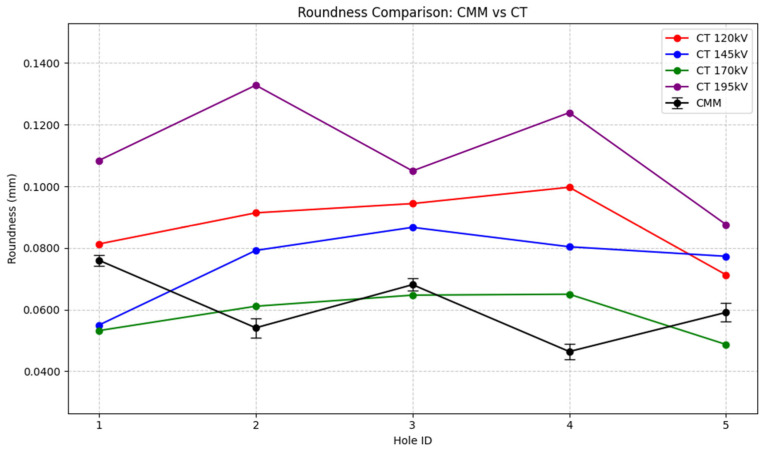
Comparison of the roundness deviation (mm) obtained from the CMM vs. the CT scans.

To provide additional insight, a graph comparing the roundness values of the holes in both techniques was created. The results are shown in Figure 17:

Examining the graph in Figure 17, it became obvious that the values of the 195 kV CT scan were consistently higher compared to the ones of the other voltage levels. However, the same happens as in Figure 16, the obtained values remain below voxel size. Again, showing the resolution limitation for detecting such small feature variations.

**Figure 18:**
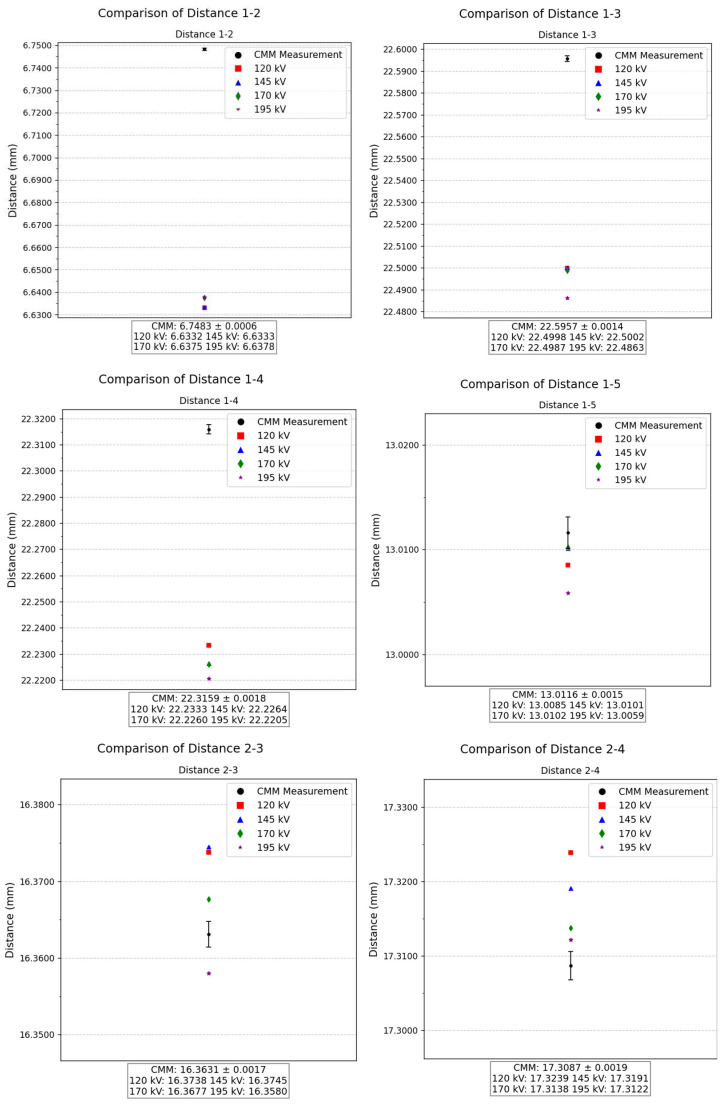

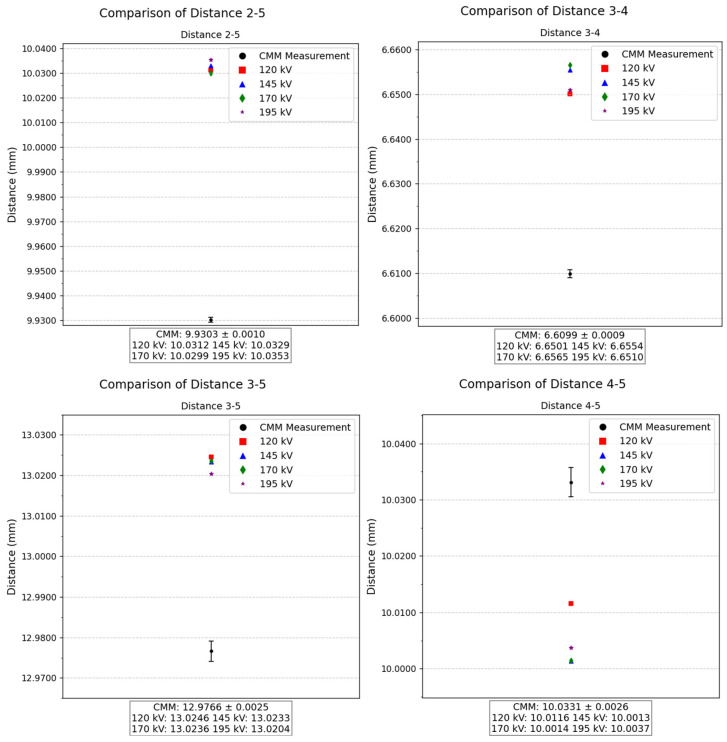
Comparison of the distances between hole centers (mm) obtained from the CMM vs. the CT scans.

Regarding the distances between hole centers, measurements were performed using both CT and CMM techniques for all combinations between holes. A total of 10 distances were analyzed, covering combinations such as 1–2, 1–3, 1–4, 1–5, 2–3, 2–4, 2–5, 3–5, and 4–5. For each case, an individual plot was created in the Figure 18 showing the CMM reference and corresponding CT measurements, allowing direct comparison of deviations.

The analysis of the 10 measured distances reveals no consistent trend in the comparison between the CMM and CT techniques. In some cases, such as the distance between 1–5 holes, the difference between both techniques is on the order of 1 µm. Nevertheless, the difference of the two techniques reaches approximately 100 µm in the case of the distance between 1–2 holes. Despite these variations, the differences between CT measurements remain stable, never exceeding approximately 10 µm across all cases. This pattern indicates that the CT-based measurements maintain a high level of consistency.

## 4. Discussion

After obtaining the experimental results from the tests, in this section these results are analyzed regarding the influence of different input parameters on CT image quality and system efficiency. In the first place, the lack of influence of RPS and steps parameters on image quality (Figure 9) is highlighted. The impact of these parameters on image quality metrics remains minor, as evidenced by the standard deviations observed in Figure 9. In Figure 9a, the CNR, SNR, and Q exhibit standard deviations of 0.018, 0.033, and 0.005, respectively. Similarly, in Figure 9b, the standard deviations are 0.043 for CNR, 0.011 for SNR, and 0.018 for Q. These low variations indicate that the influence of RPS and steps on image quality is minimal compared to other parameters such as voltage.

This behavior can be attributed to the physical limitations of the reconstruction process. The rotational step displacement produced by each step is of similar magnitude to the reconstructed voxel size of the tomographic data. Specifically, in the configuration with 360 steps, which represents the scenario with the largest displacement per step, the displacement value reaches 0.262 mm in the outer region of both ROIs. Since this displacement is comparable to the voxel dimensions of 0.155 mm, the volume generation algorithm cannot discern notable differences during reconstruction, as the incremental changes are equivalent to the spatial resolution of the data. For configurations with higher step counts, the displacement per step decreases further, reaching values as low as 0.066 mm, which is significantly below the system’s voxel size. This explains why increasing the number of steps does not lead to improved image quality. This limitation is particularly relevant in micro-CT and nano-CT systems, where smaller voxel sizes make step displacement effects more pronounced, requiring careful optimization of scanning parameters. It is expected that lowering the number of steps would result in a decrease in image quality, which may be slightly inferred from the CNR values in Figure 9b, as the displacement would become considerably bigger than the voxel size.

The voltage parameter, however, has demonstrated a significant impact on image quality, as shown in Figure 10. It may be seen that, even though every metric’s value increases with voltage, each metric reacts in slightly different ways when subjected to voltage variations. CNR is the parameter that exhibits the highest sensitivity to voltage changes, particularly at the lower end of the voltage range used in the tests. This enhanced sensitivity at lower voltages implies that the initial increases in voltage (95 to 145 kV) provide the most substantial improvements in image contrast and noise reduction. The decreasing rate of improvement at higher voltages (145 to 195 kV) indicates an approach to an optimal voltage range for the specimen under study. The SNR parameter shows a similar pattern, reaching a plateau after the initial voltage increase from 95 to 120 kV, suggesting that optimal signal-to-noise performance can be achieved at relatively lower voltage levels. As mentioned before, Q metric is the less sensitive metric out of the three analyzed metrics.

The analysis of CNR evolution with voltage (Figure 12) provided the most decisive evidence for optimizing CT parameters. The CNR improvements were substantial in the lower voltage transitions: 17.273% for 95–120 kV and 9.379% for 120–145 kV. In contrast, higher voltage transitions showed minimal gains of approximately 1.45% (for both 145–170 kV and 170–195 kV). These results clearly indicate that the higher voltage levels used in the trials approached the optimal range for maximizing CNR performance for the specimen under study. This saturation effect is attributed to the physical properties of the scanned specimen. Every object scanned in XCT has an accumulated thickness that the X-ray beam must penetrate, which depends on the applied tube voltage. As the voltage increases, the X-rays gain greater penetration power, allowing more material to be traversed effectively. However, once the voltage reaches a level sufficient to fully penetrate the sample, further increases provide only marginal improvements, as all regions of interest are already well-defined in the reconstructed image. Increasing the voltage results in a higher kinetic energy of the photons, which improves the material’s penetration capacity. Higher energy photons are less likely to be absorbed via the photoelectric effect, allowing them to travel through a greater thickness of material. The dominant interaction at higher energies is Compton scattering, where photons transfer part of their energy to outer-shell electrons, allowing the photons to continue through the material with reduced energy. Additionally, Rayleigh scattering plays a minor role, where photons are scattered without losing energy, but this interaction does not significantly affect penetration. Beyond a certain voltage, further increases in voltage lead to only slight improvements, as the material has already been adequately penetrated, resulting in a plateau in the CNR curve. This suggests that the optimal scanning voltage for the specimen has been reached, and additional energy input does not significantly enhance image quality. This finding was combined with energy efficiency considerations at 195 kV. At this voltage, shorter exposure times resulted in comparable overall energy consumption while maintaining the highest quality metrics. These factors positioned this voltage level as the optimal operating point when combined with reduced exposure times.

Regarding energy efficiency, the analysis of power consumption and exposure time (Figure 13) revealed important relationships. Higher voltages increased the unit power consumption. However, they significantly reduced the required exposure time. This reduction resulted in lower overall energy consumption. For instance, at 195 kV with 360 steps and 8 RPS, the energy consumption was only 2.762 kWh compared to 7.106 kWh for the same configuration at 95 kV, representing a reduction of approximately 61% in energy usage. This finding is very interesting in terms of CT industrial operation optimization. That may be better explained by the relationship between CNR and energy consumption at different voltage levels (Figure 14). While higher energy consumption per voltage increment does yield quality improvements, these gains are relatively modest. This implies that operating at shorter exposure times should generally prove beneficial with respect to productivity while not critically undermining image quality. Substantial differences may be found, by contrast with quality, in the values of energy consumption when increasing exposure time, as it may be seen in 145 kV scans, where CNR values vary from 7.605 to 7.669 while energy consumption values range from 3.043 kWh to 29.796 kWh. However, it is worth noting that selecting the absolute minimum exposure time may not always be necessary, because the middle points on each voltage curve could offer an acceptable balance between quality and efficiency.

Concerning the results obtained from the dimensional and geometrical measurements performed in CMM and CT technologies, several findings were made. The diameter measurements showed that CT scans displayed consistent trends across different scanning voltages, with values closely aligned to the CMM ones. Nevertheless, after calculating the bias between both techniques, it was observed that the variations remained below CT scan voxel size, suggesting that the resolution used lacks sensitivity to detect the features examined in the heat exchanger.

An interesting observation regarding hole 5 is that the cylinder wall thickness is greater compared to the thickness of the other 4 cylinders. This variation potentially explains the consistently larger diameter values observed across all scanning voltages in hole 5, as the structural variation might have influenced the penetration capability of the X-rays.

Roundness measurements inferred the same conclusion as before. It may be possible that the 195 kV scan values are higher due to the improved image clarity demonstrated before, as it could enable a better detectability of surface roughness. Anyways, the values remain below 0.155 mm voxel size, addressing the current resolution’s limitation to detect the variations of the current sample. As shown in Figure 16, despite a substantial 62.5% increase in voltage from 120 kV to 195 kV, the observed dimensional variations remain within a range smaller than 25% of the voxel size. This suggests that, while voltage adjustments influence imaging parameters, their impact on dimensional accuracy is considerably less pronounced compared to their effect on image quality metrics, at least for this scenario. Consequently, improvements in sharpness or contrast due to higher voltage do not necessarily translate into significant enhancements in metrological precision, as the fundamental resolution limitation imposed by voxel size remains the dominant factor.

Future research could involve transitioning to micro-CT or nano-CT techniques in order to develop dimensional and geometrical study, as these techniques would be sensitive to data variations.

## 5. Conclusions

To sum up, a list of conclusions has been extracted from the research carried out:

- The results underscore that in the context of X-ray tomography for non-destructive inspection applications, some parameters have been found not to alter the quality of the resulting images significantly. These mentioned parameters are the radiographies taken per step and the total number of steps during the scan. However, it is imperative to acknowledge that these very same parameters do indeed have a significant impact on energy consumption levels.
- The independent analysis of the trials conducted at each voltage level reveals that as energy consumption increases, the resulting improvement in quality metrics is marginal, exhibiting an incremental CNR enhancement on the order of 0.0025 per additional kWh consumed.
- In all the experiments conducted, one noteworthy finding is that image quality and energy consumption are inversely related. The results prove that lower voltage levels require longer exposure times to achieve comparable quality. This underlines the importance of finding an appropriate compromise between the image quality sought and the energy that is to be consumed in the process.
- The results clearly emphasize that voltage is the most important scanning parameter, since increasing the penetration power results in much better-quality levels. Nonetheless, once the necessary penetration value for the sample being assessed is reached, the additional quality improvements become minimal, reaching a plateau.
- Regarding dimensional measurements, the current CT scanning resolution has been concluded to be insufficient for detecting the geometric variations of the sample under study. Despite voltage variations of up to 62.5%, dimensional deviations remained within a range smaller than 25% of the voxel size, reinforcing the resolution limitation as the dominant factor in metrological precision.
- This study has some limitations. The lack of further statistical analysis and real-world validation using alternative CT setups may impact the generalizability of the findings. Additionally, conclusions are based on a specific material and geometry, limiting direct applicability to other cases. While the findings provide valuable insights into CT parameter optimization for a representative additive manufacturing component, future work could benefit from extended statistical analysis and validation across different CT systems and configurations.
- Future research should explore a wider range of materials, geometries, and input parameters. Higher resolution techniques such as micro-CT or nano-CT could refine results. AI-driven optimization of scan parameters is another promising approach to enhance image quality and energy efficiency. However, AI requires large datasets, often synthetic due to the high cost of real inspections. Generating reliable synthetic CT data remains challenging due to multiple sources of error in the CT process. Experimental studies like the one presented here are essential for advancing AI-based research in this field.

## Figures and Tables

**Table 1:** Mean values and standard deviations (Std. Dev.) of CNR, SNR, Q metric, and equipment P (W) for each parameter combination, based on voltage variations.

Test ID	Average CNR	Std. Dev. CNR	Average SNR	Std. Dev. SNR	Average Q	Std. Dev. Q	Average P (W)	Std. Dev. P (W)
1–9	7.857	0.020	7.355	0.031	3.611	0.005	27.666	0.447
10–18	7.745	0.019	7.382	0.280	3.573	0.004	25.892	0.129
19–27	7.633	0.029	7.348	0.032	3.536	0.012	25.216	0.273
28–36	6.979	0.038	7.342	0.024	3.305	0.028	23.528	0.220
37–45	5.951	0.074	5.708	0.044	2.479	0.034	22.506	0.245

**Table 2:** Results of the CMM measurements: diameter (mm), roundness deviation (mm) and their average and standard deviation values (mm).

Hole ID	Average D (mm)	D Std. Dev. (mm)	Average Rd. Dev. (mm)	Rd. Dev. Std. Dev. (mm)
1	2.8398	0.0015	0.076	0.0018
2	2.8410	0.0047	0.054	0.031
3	2.8584	0.0029	0.068	0.0020
4	2.8418	0.0036	0.046	0.0025
5	2.8662	0.0034	0.059	0.0030

**Table 3:** CT results of the diameter (mm) measurements for each hole.

Test ID	Hole 1 D (mm)	Hole 2 D (mm)	Hole 3 D (mm)	Hole 4 D (mm)	Hole 5 D (mm)
5	2.7948	2.8214	2.8262	2.807	2.8562
14	2.8762	2.867	2.8464	2.855	2.8982
23	2.856	2.859	2.8302	2.8356	2.8678
32	2.8556	2.8496	2.8262	2.8396	2.8864
41	-	-	-	-	-

**Table 4:** CT results of the roundness deviation (mm) measurements for each hole.

Test ID	Hole 1 Rd. Dev. (mm)	Hole 2 Rd. Dev. (mm)	Hole 3 Rd. Dev. (mm)	Hole 4 Rd. Dev. (mm)	Hole 5 Rd. Dev. (mm)
5	0.1084	0.1328	0.105	0.1239	0.0876
14	0.0532	0.0611	0.0647	0.065	0.0487
23	0.055	0.0792	0.0867	0.0804	0.0773
32	0.0813	0.0914	0.0944	0.0997	0.0713
41	-	-	-	-	-

## Data Availability

All data supporting the reported results are included within the article.
